# Evaluation of a prototype Orbitrap Astral Zoom mass spectrometer for quantitative proteomics – Beyond identification lists

**DOI:** 10.1101/2025.05.30.657132

**Published:** 2025-06-25

**Authors:** Chris Hsu, Nicholas Shulman, Hamish Stewart, Johannes Petzoldt, Anna Pashkova, Deanna L. Plubell, Eduard Denisov, Bernd Hagedorn, Eugen Damoc, Brendan X. MacLean, Philip Remes, Jesse D. Canterbury, Alexander Makarov, Christian Hock, Vlad Zabrouskov, Christine C. Wu, Michael J. MacCoss

## Abstract

Mass spectrometry instrumentation continues to evolve rapidly yet quantifying these advances beyond conventional peptide and protein detections remain challenging. Here, we evaluate a modified Orbitrap Astral Zoom mass spectrometer (MS) prototype and compare its performance to the standard Orbitrap Astral MS. Across a range of acquisition methods and sample inputs, the prototype instrument outperformed the standard Orbitrap Astral MS in precursor and protein identifications, ion beam utilization, and quantitative precision. To enable meaningful cross-platform comparisons, we implemented an ion calibration framework that converts signal intensity from arbitrary units to ion per second. This benchmarking strategy showed that the prototype sampled 30% more ions per peptide than the original Orbitrap Astral MS. This increase in the ion beam utilization resulted in improved sensitivity and quantitative precision. To make these metrics broadly accessible, we added new metrics to the Skyline document grid to report the number of ions measured in a spectrum at the apex of the elution peak or the sum of ions between the peak integration boundaries. Taken together, our results demonstrate the prototype Orbitrap Astral Zoom as a high-performance platform for DIA proteomics and establish a generalizable framework for evaluation of mass spectrometer performance based on the number of ions detected for each analyte. Data are available on Panorama Public and on ProteomeXchange under the identifier PXD064536.

## Full Text Availability

The license terms selected by the author(s) for this preprint version do not permit archiving in PMC. The full text is available from the preprint server.

